# Taking control: Is job crafting related to the intention to leave surgical training?

**DOI:** 10.1371/journal.pone.0197276

**Published:** 2018-06-01

**Authors:** Luis Carlos Dominguez, Laurents Stassen, Willem de Grave, Alvaro Sanabria, Edgar Alfonso, Diana Dolmans

**Affiliations:** 1 Department of Surgery, Universidad de la Sabana, Chía, Colombia; 2 Department of Surgery, Maastricht University Medical Center (MUMC+), Maastricht, the Netherlands; 3 Department of Educational Development and Research, School of Health Professions Education (SHE), Faculty of Health, Medicine and Life Sciences (FHML), Maastricht University, Maastricht, the Netherlands; 4 Fundación Colombiana de Cancerología—Clínica Vida, Medellín, Colombia; 5 University of Lyon, University Jean Monnet-St-Etienne, LASPI, Saint-Etienne, France; Indiana University, UNITED STATES

## Abstract

**Objective:**

The intention to leave surgical training, hereinafter referred as proxy of “attrition,” is associated with poor well-being in the workplace. Attrition is suggested to diminish when residents possess job-crafting skills, that is, the ability to redefine their job in meaningful ways and maximize well-being at work by increasing structural and social resources and challenges and decreasing hindering demands. However, the evidence supporting this relationship is scant. This study sought to: 1) investigate to what extent residents possess job-crafting skills and compare residents’ levels of job-crafting skills across years of residency training; 2) investigate the relationship between job crafting, well-being as measured by burnout and work-engagement rates, and the intention to leave; and 3) compare the levels of job-crafting skills and well-being between residents with and without serious intentions to leave.

**Methods:**

This cross sectional study was conducted in fifteen residency programs in Colombia. Surgical residents completed different questionnaires including the Dutch Job Crafting Scale (DJCS), MBI-Human Services Survey (MBI-HSS), Utrecht Work Engagement Scale (UWES-17) and an adapted version of the Nurse Turnover Intention Scale (NTIS). The objectives were addressed by independent analyses of variance (ANOVA), structural equation modeling techniques (SEM) and independent t-tests, respectively.

**Results:**

A total of 202 residents participated. Residents generally scored high on their job-crafting skills to increase structural and social resources as well as challenging demands, but were less positive about their skills to reduce hindering demands. No differences across years of training were found. Job crafting correlated positively with work-engagement, which was inversely related to the intention to leave. Conversely, job crafting correlated negatively with burnout, which bore a positive relationship to the intention to leave. Residents *with* serious intentions to leave exhibited lower levels of most job-crafting skills and work-engagement, compared to those *without* such intentions.

**Conclusions:**

This study adds evidence that attrition is a process mediated by residents’ well being at work, which can be molded by their job-crafting endeavors. Future research is needed to evaluate the effectiveness of interventions aimed at cultivating resident’s job-crafting abilities in order to reduce attrition.

## Introduction

Attrition in surgery is a serious issue, with 20% of residents dropping out, and an even greater proportion, some 32% to 58%, having serious intentions to leave the program [1,2,3]. Attrition has negative consequences not only for individuals, but also for education and healthcare systems, representing significant losses of resources and time for training [4,5]. Additionally it is associated with difficulties to replace residents and workforce shortages of future surgeons [2,6,7]. Recent evidence indicates that attrition is more closely associated with a poor state of well-being at work than with demographic and individual factors, which seems plausible as learning takes place almost exclusively in a workplace-based environment [2,3,5,6].

Excessive training demands and related lifestyle issues, nurture this poor state of well-being [2,8,9,10,11], which may lead to burnout, a state characterized by emotional exhaustion, depersonalization and lack of personal accomplishment, ultimately associated with serious thoughts of leaving training [12,13,14,15]. A recent U.S. publication revealed for instance that, of the surgical residents who considered leaving the residency (44%), a majority met the criterion for burnout [16]. The work environment, however, can also have a moderating effect on attrition, which is the case when levels of work engagement are high. Work engagement is a state opposite to burnout characterized by vigor, dedication, and absorption [12], which foster satisfaction, quality of life and resident’s retention in surgical training [17,18,19].

Although residents fulfill a dual role as trainees and workers in clinical environments, the process of attrition may be considered similar to that of leaving a job, in which the strongest predictor is the intention to leave, defined as the conscious and deliberate willingness to leave the job [20,21]. Such intentions are mediated, inter alia, by the individual’s well-being at work, attitude, and expectations about the organizational turnover, and by subjective norms and social pressure [22,23]. In surgical training, two approaches have been proposed to improve working conditions and reduce such intentions among residents: 1) to restrict the number of weekly working hours, the impact of which on attrition has been minimal [4,24,25,26]; and 2) to promote residents’ abilities to transform working conditions by themselves [2,27,28]. The latter approach relates to the idea of “job crafting,” that is, employees’ ability to strategically balance demands and resources at work on the one hand with their personal abilities and needs on the other [29]. Job crafting is a type of proactive person-environment (PE) fit behavior, oriented to achieve a better fit between one’s own attributes and that of the internal work-environment [30]. Wrzesniewski and Dutton introduced the concept to explain the physical and cognitive changes that individuals make in the task or relational boundaries at work [31]. The aims of job crafting are to take control, to create a positive self-image and to connect to others. These aims are attained by task, relational and cognitive crafting [31]. In doing so, employees take control to increase personal satisfaction, improve their relationships with others, extend their knowledge about work avoiding negative consequences, and participate actively in job design [31,32]. However, there is little evidence that this approach will actually reduce attrition in surgery.

The current concept of job crafting is embedded in the Job Demands–Resources (JD-R) theory, a dynamic model used to understand, explain, and predict well-being in terms of burnout and work engagement rates, their causes, and performance outcomes, irrespective of the occupational setting [12,33]. Within JD-R theory, job crafting encapsulates a set of four skills, specifically the ability to: (1) increase structural resources (those that promote responsibility, autonomy, and/or knowledge about work), (2) increase social resources (those that promote socialization, such as feedback and coaching, and satisfactory interactions with others, such as social support and collaboration), (3) increase challenging demands (those that promote personal growth and personal achievement), and (4) decrease hindering demands (those circumstances that involve excessive or undesirable constraints that interfere with or inhibit the achievement of valued goals, such as role conflict and workload) [12,32].

In general, employees who engage in job crafting have been demonstrated to perform better, as they are more engaged and less prone to burnout [34,35]. Whether this also holds true for trainees who learn by working in educational contexts such as medical residencies, however, has not yet been sufficiently investigated. Similarly, the impact of trainees’ job-crafting endeavors on training outcomes has received scant attention. Most studies have hitherto concentrated on trainee attributes, such as grit (defined as passion and perseverance for long term outcomes), resilience and conscientiousness, which have been found to strongly predict academic success [36,37,38,39]. What these studies failed to address, however, is how such attributes help trainees to change their own well-being at work, increasing and reducing the levels of work engagement and burnout, respectively, and influencing performance outcomes (e.g., staying in or leaving the program). A focus on job-crafting skills in trainees may help fill this gap. Unfortunately, it still remains inconclusive whether job crafting is a skill that surgical residents automatically develop over time, as a mechanism to control the continuous changes in demands and resources in the workplace. We expect job-crafting skills to be instrumental in such workplace-based learning environments, for training outcomes depend not only on efforts of faculty members to create a good training climate [40], but also on residents, who in daily practice must cope with tensions between service delivery and education. In fact, the decision to leave a program may largely depend on residents’ ability to control and effectively change the work environment and, consequently, to find purpose and meaning in the residency training. In sum, the surgical realm needs in-depth knowledge about the role of job-crafting skills and its related practical implications for continuing professional development and support of residents. This may also enhance our understanding of the processes involved in residents’ retention because job crafting is a mediator between both, situational and personal predictors, and work engagement at the individual and organizational levels [41,42]. In practice, this perspective could be input for the call for research about attrition in surgical training from a positive side [43].

The purpose of this study is therefore to investigate the relationship between job crafting in surgical training and the intention to leave the program. To this end, we will address the following research questions (RQ) and hypotheses (H):

**RQ 1:** To what extent do surgical residents possess job-crafting skills and does this differ across the years of surgical training?

The main hypothesis underpinning this question is:

H1: Job-crafting skills differ significantly across years of training
**RQ 2:** What are the relationships between job-crafting skills, well-being at work (as measured by work engagement and burnout), and the intention to leave the program in surgical residents?

**Fig 1:**
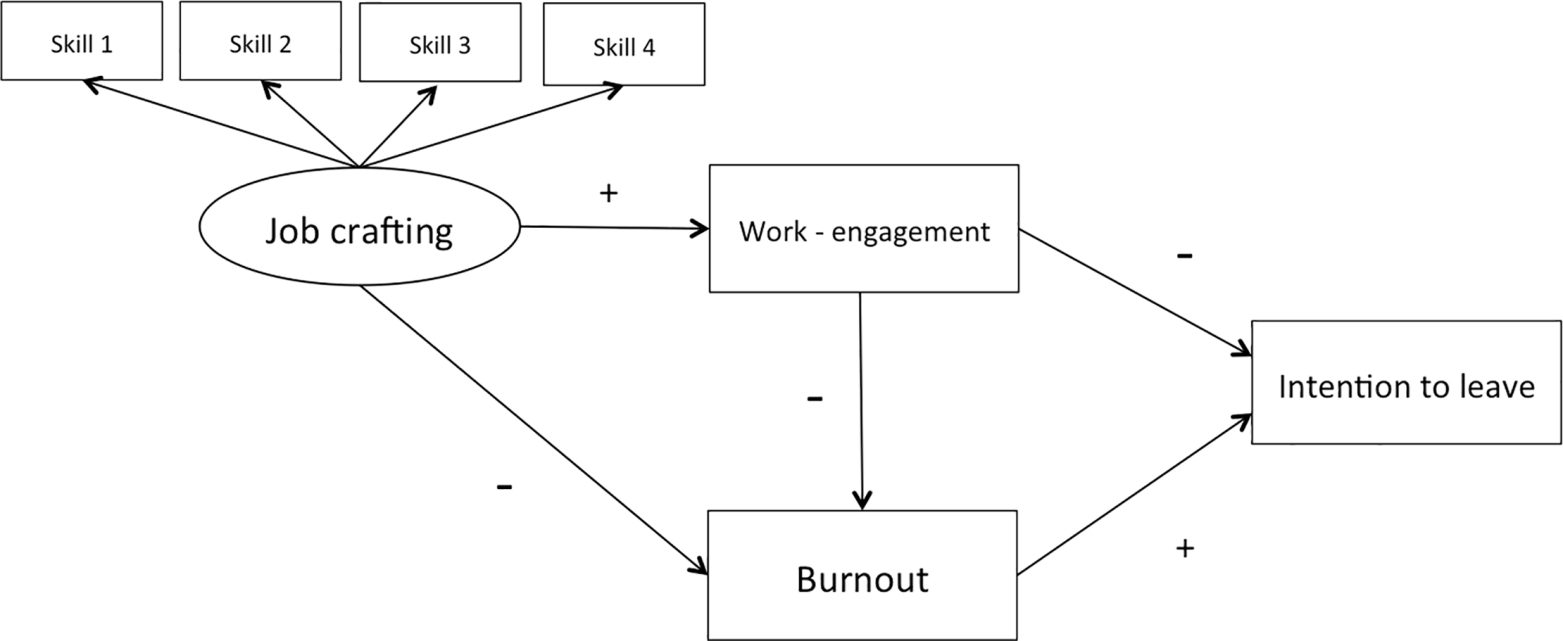
Theoretical model. Skill 1: the ability to increase structural resources. Skill 2: the ability to increase social resources. Skill 3: the ability to increase challenging demands. Skill 4: the ability to decrease hindering demands“.

In order to investigate these relationships, we incorporated them into the theoretical model presented in Fig 1. The hypotheses underpinning these relationships are:

H2: Job-crafting skills are inversely related to the intention to leave the program.
H3a: Job crafting is negatively related to burnout
H3b: Burnout is positively related to the intention to leave the program.
H4a: Job crafting is positively related to work engagement
H4b: Work engagement is negatively related to the intention to leave the program.

**RQ 3:** What are the differences in job-crafting skills and well-being (as measured by work engagement and burnout rates) between surgical residents with serious intentions to leave the program and those without serious intentions?

The main hypothesis underpinning this question is:

H5: Levels of job-crafting skills and work engagement are lower and the incidence of burnout higher among residents with serious intentions to leave the program compared to those without these intentions.

## Materials and methods

### Setting

Medical training in Colombia consists of a six-year undergraduate program that includes one internship year. Upon completion, doctors apply to private or public universities for a residency position. The surgical residency spans approximately 400 positions, which are distributed between 21 programs (2015). Residents complete four years of full-time training, and then apply for a sub-specialty program of two more years. They pay a tuition fee to the university but usually do not receive a salary from hospitals. Few residents receive a semi-annual governmental scholarship. The national policy on duty hours is 66 hours/week.

### Participants

Twenty-one programs of General Surgery were asked to participate. We sent invitations to program directors or deans of medical schools. Five programs never responded. One program rejected the invitation. Finally we invited 284 residents from 15 programs located in seven cities across the country. Based on the power analysis, the present study required a sample of 197 participants (α error = .05; β error = . 20; 95%CI).

### Research design

This is a quantitative cross-sectional study following a correlational approach. We obtained ethical approval for the present study from the Commission of Medical Education, Faculty of Medicine, Universidad de la Sabana, Colombia (Reference number: 14/2015)

### Measures and instruments

We measured job-crafting skills using a Spanish version of the Dutch job-crafting scale (DJCS) [34,44], which consists of 21 items ranging from 1 (never) to 5 (very often). The scale’s α coefficient of reliability ranged from 0.70–0.80 [34,44]. The instrument contains four subscales that each one measures one job-crafting skill:

■ Skill 1: the ability to increase structural resources (5 items). One such item read, for instance: “I make sure that I use my capacities to the fullest.”
■ Skill 2: the ability to increase social resources (5 items). For instance: “I ask others for feedback on my job performance.”
■ Skill 3: the ability to increase challenging demands (5 items). For example: “When an interesting project comes along, I offer myself proactively as project co-worker.”
■ Skill 4: the ability to decrease hindering demands (6 items). For example: “I manage my work so that I try to minimize contact with people whose problems affect me emotionally.”

We measured the intention to leave using an adapted version of the Nurse Turnover Intention Scale (NTIS) [45]. Because the scale was not available in Spanish language, we translated it from English into Spanish, adhering to the procedure set out in the IQOLA recommendations [46]. The scale consists of three items ranging from 1 (never) to 5 (very often/likely). It has a proven α coefficient of 0.85 [45]. One of these items read: “How often did you generate the idea to leave the surgery residency in the past six months?”

To gauge work engagement we used a Spanish version of the Utrecht Work Engagement Scale (UWES-17) [47], which comprises 17 items ranging from 0 (never) to 6 (always-every day). It reportedly has a Cronbach’s alpha of 0.88–0.95. An example of such an item is: “I find the work that I do full of meaning and purpose.” Finally, we used a Spanish version of the MBI-Human Services Survey (MBI-HSS) to quantify burnout [48]. The scale consists of 22 items ranging from 0 (never) to 6 (always-every day) distributed in three domains (emotional exhaustion, depersonalization and personal accomplishment). One of these items read, for example: “I feel frustrated by my job”. Burnout results from a combination of high levels of emotional exhaustion with high levels of depersonalization or low levels of personal accomplishment [49]. The scale’s α coefficients of reliability ranged from 0.71–0.90 [48].

### Procedure

We prepared a paper-based questionnaire for data collection. After we had pilot tested and subsequently adjusted the instruments, we contacted the program directors and/or deans of the medical schools in order to explain the purpose of the study. When they voluntarily agreed to participate, we organized different visits to surgical meetings in ten programs, which all residents regularly attended (usually once a week). There we informed residents of the study’s purpose, anonymity, confidentiality, and further management of information. In the remaining five programs, the program directors arranged the collection of information. We deliberately did not distribute the instrument during clinical activity, nor did we give participants any financial compensation or any other type of incentive. We collected data in the period spanning October to December, 2015. Once completed, the main researcher coordinated the transcription and organization of data in order to guarantee the quality and integrity of information.

### Statistical analysis

Resident questionnaires that exceeded 50% missing data were excluded from further analyses. On the contrary, questionnaires with less than 50% missing data were imputed by the expectation-maximization technique. Frequencies and descriptive statistics were measured to gain insight in relevant study sample characteristics. We calculated the mean, standard deviation and ranges of each domain of burnout. The cut-offs for high, moderate and low levels of each domain were calculated according to the standard recommendations [49]. The rate of burnout was reported as percentage.

To test H1, we computed the mean scores and standard deviations (SD) for each skill on the DJCS, as criterion variables. We defined that scores of 4.0 or higher were considered as good, between 3.0 and 4.0 as sufficient, and below 3.0 as low (scale 1–5). We then performed univariate analyses of variance (ANOVA) for each skill, considering the year of residency as predictor variable. In each ANOVA we calculated the sum of squares (SS) as a crude measure of variability, the mean square (MS) as variance estimate, and the mean square error (MSE) as measure of error variance. The differences for each ANOVA were considered significant when *P* < .05. The proportion of variance in each skill accounted for by the year of residency was calculated by the omega squared (ω2) as index of effect size. Following the available recommendations, a ω2 = .01 represents a small effect, ω2 = .06 a medium effect, and ω2 = .14 a large effect [50].

We tested the hypothetical relationships expressed in H2 through H4b and represented as a model in Fig 1 using structural equation modeling (SEM). We applied SEM analyses because these are highly flexible statistical procedures that allow researchers to test theoretical models by analyzing correlational data [51]. SEM is useful because it provides information with regards to hypothesized relationships in a sample of participants. SEM informs about how the theoretical model fits against the empirical data, with the aims to inform logical and consistent relationships between independent, mediator and dependent variables. However, SEM does not provide cause and effect, like the evidence provided by an experiment [51].

For SEM analyses, we initially established convergent validity of the model based on inter-scale correlations (satisfactory if < .70; significant when *P* < .05). Also, we calculate the coefficients of reliability for each scale. The internal consistency of scales was considered appropriate if the α coefficient of reliability was >.70. Next, we followed a model-fit procedure to test the whole hypothesized model against the empirical data by using the method of maximum likelihood of estimation. We selected this method given the fixed number of observations in the sample and its normal distributions. The free parameters (those estimated from the data) were non-standardized. We assessed model fit based on the standardized root mean square residual (SMRS), root mean square error of approximation (RMSA), comparative fit index (CFI), and Tucker-Lewis index (TLI). The cut-offs for good fit were: SMRS p < .08 for good fit and < .12 for acceptable fit, RMSA < .06 for good fit and < .10 for acceptable fit, and CFI and TLI >.95 for good fit and >.90 for acceptable fit [51].

Additionally, we conducted mediation analyses to measure the indirect and direct effects of job crafting, work engagement and burnout on the intention to leave surgical training in the theoretical model. The indirect effect of one variable on a second variable is defined as the part of the total effect that is transmitted by one o more mediating variables in the model [51]. In the present study we measured two indirect effects. First, we estimated the indirect effect of job crafting on the intention to leave mediated by burnout and work engagement. Second, we estimated the indirect effect of work engagement on the intention to leave mediated by burnout. On the other hand, the direct effect is defined as the part of the total effect that is not mediated by another variable [51]. In the present study we measured two direct effects. First, we estimated the direct effect of work engagement on the intention to leave not mediated by burnout. Then, we estimated the direct effect of burnout on the intention to leave not mediated by work engagement and job crafting. The effects were considered significant when p < .05. Finally, we informed the unstandardized path coefficients (b coefficients) for any path between the independent and the dependent variables included in the model (p < .05).

To test H5, we grouped our study population into residents *with* (Group 1) and residents *without* (Group 2) serious intentions to leave the program. We defined such intentions based on participants’ scores for the three items on the NTIS: those with high scores (4: often/likely; and 5: very often/likely) were assigned to Group 1; the remainder to Group 2. For each group we calculated the mean scores and SD per skill. We conducted independent t-tests to determine whether the mean scores per skill of Group 1 were significantly different from those of Group 2 (*P* < .05). We computed *Cohen’s d statistic* to indicate the size of the difference between means for each variable in both groups (95%CI). The reference criteria for interpreting the d statistic were: small size d = ± .20, medium size d = ± .50, and large size d = ± .80 [52]. Finally, we calculated the differences in the incidence of burnout between both groups using the chi-squared test (χ^2^) (p < .05). We performed the statistical analysis using Stata-13 software.

## Results

A total of 202 surgical residents (men: 63.9%) agreed to participate, denoting a response rate of 71.1%. Residents were aged between 23 and 41 years old, with a mean age of 28.63 (SD: 2.96). One hundred and thirty-one of these residents (64.9%) were from private programs. The number of participants per program ranged from 6 to 23 residents. The main characteristics of participants are described in Table 1.

**Table 1:** Characteristics of the study population.

Characteristics	Study sample
Number of residents	202
Male residents, n	129
Female residents, n	73
Number of training programs evaluated, n	15
Number of public programs, n	6
Number of private programs, n	9
Number of residents per public programs, n	71
Number of residents per private programs, n	131
**Number of residents per years of residency training, n**	
1	67
2	43
3	45
4	47

### Descriptive findings

The mean score and SD (95%CI) for intention to leave was 1.39 (.59) (1.30–1.47). The scores for job crafting skills were: ability to increase structural resources (skill 1) = 4.50 (.34) (4.45–4.55); ability to increase social resources (skill 2) = 3.86 (.68) (3.76–3.95); ability to increase challenging demands (skill 3) = 3.39 (.71) (3.29–3.49); ability to decrease hindering demands (skill 4) = 2.71 (.14) (2.61–2.82). Hence, residents generally scored high on their job-crafting skills to increase structural and social resources as well as challenging demands, but were less positive about their skills to reduce hindering demands. The score for work engagement was 5.38 (.47) (5.31–5.45). The global percentage of burnout was 33.2 (67 residents). Half of the residents scored high on emotional exhaustion, 30% high on depersonalization and 41,6% low on personal accomplishment. The mean scores and the distribution of residents in each domain of burnout are presented in Table 2.

**Table 2:** Burnout among surgery residents (n = 202). Mean scores (SD and ranges) per domain and percentage of residents in each domain.

Domain	Mean score, SD, range	Low (%)	Moderate (%)	High (%)
Emotional Exhaustion	28.11 (10.23) (5–52)	14	29.7	55.4
Depersonalization	10.35 (6.36) (0–28)	44.1	25.7	30.2
Personal accomplishment	35 (4.81) (17–42)	41.6	40.1	18.3

SD: standard deviations

### Results from SEM analyses

The descriptive statistics, inter-scale correlations, and reliability estimates are displayed in Table 3. Correlations ranged from -0.004 to 0.40, satisfying the recommended criterion (<0.70). Moreover, the model showed adequate fit to the empirical data: SMRS = .05; RMSA = .065; CFI = .93 and TLI = .87.

**Table 3:** Correlations, descriptive statistics and coefficients of reliability (on the diagonal) for the included variables.

Variable (Scale)	M	SD	1	2	3	4	5	6	7
Skill 1: ability to increase structural resources (DJCS) (a) (1)	4.50	.34	(0.74)						
Skill 2: ability to increase social resources (DJCS) (a) (2)	3.86	.68	.19**	(0.76)					
Skill 3: ability to increase challenging demands (DJCS) (a) (3)	3.39	.71	.27**	.39**	(0.82)				
Skill 4: ability to reduce hindering demands (DJCS) (a) (4)	2.71	.74	.10	.15*	.28**	(0.81)			
Intention to leave (NTIS) (b) (5)	1.39	.59	-.19**	-.13	-.13	.14*	(0.65)		
Work engagement (UWES-17) (c) (6)	5.38	.47	.28**	.21**	.35**	.12	-.40**	(0.80)	
Burnout (MBI-HSS) (c) (7)	-	-	-.07	-.16*	-.20**	-.0004	.36**	-.31**	(0.85)

N = 202

* *P* < .05

** *P* < .01. Dutch Job-Crafting Scale (DJCS); Nurse Turnover Intention Scale (NTIS), Utrecht Work Engagement Scale (UWES); Maslach Burnout Inventory—Human Services Survey (MBI-HSS); M = Mean; SD = Standard Deviation. a) Scale of 1–5, 1 = Never, 5 = Very often. b) Scale of 1–5, 1 = Never, 5 = Very often/likely. c) Scale of 0–6, 0 = Never, 6 = Always/Every day.

**Fig 2:**
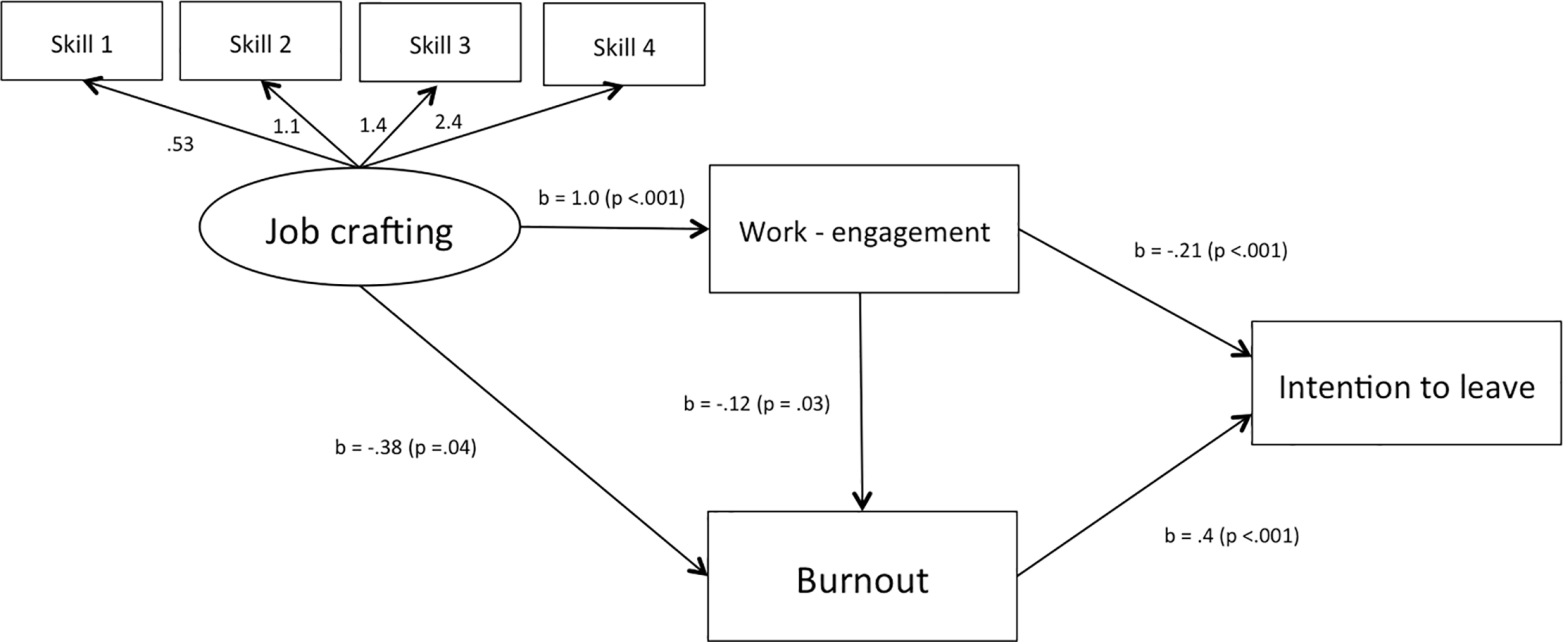
Theoretical model (including path coefficients and p values). Skill 1: the ability to increase structural resources. Skill 2: the ability to increase social resources. Skill 3: the ability to increase challenging demands. Skill 4: the ability to decrease hindering demands.

Considering the proposed model, the results of mediation analyses indicated that 100% of total effects of job crafting on the intention to leave surgical training were indirect effects (p < .05). In other words, the total effects of job crafting were mediated by burnout and work engagement. The indirect effect of job crafting via burnout was -.19 (48,7%) (p < .05). In turn, the indirect effect of job crafting via work engagement was -.20 (51,7%) (p < .05). These results confirmed that both mediating variables (burnout and work-engagement) transmitted the total effect of the first variable (job crafting) on the second variable (intention to leave). On the other hand, 18.4% of the total effects of work engagement on the intention to leave were indirect effects. The indirect effect of work engagement via burnout was -.047 (p < .05). Conversely the direct effect of work engagement on the intention to leave was -.20 (p < .05), corresponding to 81.6% of the total effects. Finally, 100% of the total effects of burnout on the intention to leave were direct effects (.39) (p < .05). Fig 2 represents the model for the intention to leave surgical training with unstandardized path coefficients.

### Test of hypotheses

H1: Job-crafting skills differ significantly across years of training

The levels of skills 1 to 3 were moderately high for all years of training, but those of skill 4 remained consistently low. Based on ANOVAs, we found non-significant differences in level of skills between the years of training (p < .05). The proportion of variance (ω2) presented small effect sizes for all skills accounted for by the year of residency. These results are presented in Table 4. We therefore reject H1.

**Table 4:** Results of univariate ANOVA for job-crafting skills (in each ANOVA the predictor variable is the year of residency).

Criterion Variable	Means and Confidence Intervals for each year of residency								ANOVA							
	Year 1 (n = 67)		Year 2 (n = 43)		Year 3 (n = 45)		Year 4 (n = 47)		SS	df	MS	MSE	F	p	Effect size	
	M	[95% CI]	M	[95% CI]	M	[95% CI]	M	[95% CI]							ω2	95%CI
Skill 1: Ability to increase structural resources	4.50	(4.41–4.58)	4.47	(4.37–4.57)	4.53	(4.44–4.63)	4.50	(4.39–4.62)	15.35	12	1.27	11.70	.93	.51	0	0 - .007
Skill 2: Ability to increase social resources	3.95	(3.79–4.12)	3.80	(3.61–3.99)	3.78	(3.55–4.01)	3.85	(3.65–4.04)	25.40	20	1.27	11.72	.92	.55	0	0 - (-.26)
Skill 3: Ability to increase challenging demands	3.48	(3.29–3.66)	3.28	(3.08–3.47)	3.26	(3.07–3.46)	3.49	(3.26–3.72)	28.73	19	1.51	11.61	1.12	.33	.01	0 - .005
Skill 4: Ability to reduce hindering demands	2.74	(2.55–2.93)	2.57	(2.36–2.79)	2.70	(2.51–2.89)	2.82	(2.58–3.07)	31.25	24	1.30	11.71	.95	.53	0	0 - (-.40)

N = 202. M = Mean; CI = 95% Confidence Interval for the mean. SS = Sum of squares; df = degree of freedom; MS = Mean Square; MSE: Mean Square error; F = F Statistic, p <0.05; ω2 = omega squared; ANOVA = Analysis of variance; CI = Confidence Interval

H2: Job-crafting skills are inversely related to the intention to leave the program.

We found that skill 1 (increase structural resources) correlated negatively (-.19) with the intention to leave and skill 4 (reducing hindering demands) correlated positively (.14) with the intention to leave. The other two job crafting skills showed no significant correlations with the intention to leave (Table 3). Consequently, we partially accept H2, considering that only two out of four job crafting skills were related with the intention to leave.

H3a: Job crafting is negatively related to burnout; H3b: Burnout is positively related to the intention to leave the program.

The results of SEM analyses indicated that job crafting was negatively related to burnout (b = -.38, p = .04) (Fig 2). Similarly, we found negative significant correlations between two job-crafting skills and burnout (skill 2 and skill 4, which correlate respectively -.16 and -.20) (Table 3). We therefore accept H3a. In H3b burnout was positively related to the intention to leave (b = .4, p < .001) (Fig 2). Additionally, we found that burnout was negatively related to work engagement (b = -.12, p = .03). We therefore accept H3b.

H4a: Job crafting is positively related to work-engagement; H4b: Work engagement is negatively related to the intention to leave the program.

The results of SEM analyses indicated that job crafting was positively related to work engagement (b = 1.0, p < .001) (Fig 2). In turn, we found positive significant correlations between three out of four job-crafting skills and work engagement (skill 1: p < .01; skill 2: p < .01; skill 3: p < .01) (Table 3). We therefore accept H3a. In H4b work engagement was negatively related to the intention to leave (b = -.21, p < .001) (Fig 2). Thus, we accept H4b.

H5: Levels of job-crafting skills and work engagement are lower and the incidence of burnout higher among residents with serious intentions to leave the program compared to those without these intentions.

Twenty-three participants (11.9%) had serious intentions to leave the program (Group 1). This group presented lower levels of skill 1 (p < .05) and skill 3 (p < .05) compared to residents without such intentions (Group 2). Hence, we partially this part of H5. We also found lower scores for work engagement (p < .05) and higher burnout rates (p < .05) in this group. Thus, we accepted this part of H5. The differences between the two groups in means of all job-crafting skills were small. These results are presented in Table 5.

**Table 5:** Comparison of job-crafting skills between residents *with* and those *without* serious intentions to leave.

Criterion Variable	Group 1 (n = 23)			Group 2 (n = 179)			p value	Effect size (Cohen’s test)	
	M	SD	[95% CI]	M	SD	[95% Cl]		Estimate	[95% CI]
Skill 1: Ability to increase structural resources	4.36	.33	4.19–4.53	4.52	.38	4.47–4.57	.01	-.41	-.85 - .19
Skill 2: Ability to increase social resources	3.77	.69	3.49–4.05	3.87	.65	3.77–3.97	.24	-.15	-.58 - .28
Skill 3: Ability to increase challenging demands	3.02	.69	2.67–3.37	3.44	.80	3.33–3.54	. < .01	-.52	-.96 - (-.08)
Skill 4: Ability to reduce hindering demands	2.96	.71	2.56–3.36	2.68	.92	2.58–2.79	.08	.30	-.12 - .74
Work engagement	4.98	.42	4.69–5.27	5.43	.66	5.37–5.49	< .01	-.69	-11 - (-.25)
Burnout	18 (78.3%)			49 (27.4%)			< .01		

N = 202. Group 1 = Residents with serious intentions to leave; Group 2 = Residents without serious intentions to leave; M = Mean; SD = Standard Deviation; CI = Confidence Interval

## Discussion

This study sought to explore the job-crafting skills in surgical residents in relation to their well-being at work (as measured by work engagement and burnout rates) and the intention to leave the training program. A first observation was that, irrespective of the year of training, the job-crafting scores for the ability to increase structural resources were good (above 4, scale 1–5), those for the ability to increase social resources and challenging demands sufficient (between 3.0 and 4.0), and those for the ability to reduce hindering demands low (below 3.0, scale 1–5). No differences between years of training were found. A second observation was that job crafting was positively related to work engagement and negatively related to burnout, while burnout was positively and work engagement negatively related to the intention to leave. Our final observation was that residents *with* serious intentions to leave exhibited lower levels of most job-crafting skills and work engagement, compared to those *without* such intentions.

The first observation suggests that surgical residents, regardless of training year, are able to deal with the complexity of the workplace-based learning environment by crafting their ability to increase structural and social resources throughout the program. This finding indicates that job crafting is a type of person environment (PE) fit behavior well developed in surgery residents from early stages, instead of only a personal trait. The current evidence on job crafting literature supports this observation [31,41]. In this regard, residents are not only skillfully to increase structural resources, such as responsibility and autonomy, and social resources, which promote socialization and fulfilling interactions, they are also able to positively increase challenging demands although too a lesser extent, for instance starting new interesting projects, which can foster personal growth. Similar findings have been found in other realms of medical education focused in the proactive seeking behaviors that enable residents to find educational resources and diminish demands in the workplace [53,54,55]. Our findings indicated that the ability to reduce hindering demands (skill 4), however, appeared to be less present in residents from all years. This finding would suggest that the resident’s skills to reduce hindering demands are less well developed. At the present, we do not have a satisfactory explanation for this finding. Future research may seek to explain the lack of this latter ability (e.g. through the lens of resilience and spirituality frameworks) and may focus on how to increase resident’s empowerment in order to deal with hindrances at the workplace.

Our second and third observations place in context the relevance of resident’s proactivity at work to understand the intention to leave, mediated by well-being. Preliminary research in the field of medical education has a limited scope to explain the influences of proactivity on wellbeing and performance. For instance, autonomy and challenge seeking behaviors inform the proactive changes that trainees initiate to gain entrustment of professional activities, autonomy, responsibility and opportunities [53,54]. Similarly, feedback-seeking behavior describes the proactive changes oriented to increase supervision, coaching, feedback and social support [55]. Nonetheless, how these proactive seeking behaviors foster burnout and work engagement remains inconclusive. The present study adds evidence in this realm. We explored the relationships between resident’s proactivity, throughout job crafting, and performance outcomes (intention to leave), mediated by burnout and work engagement. We believe that this framework is relevant due to different reasons. Currently, surgical training requires residents not only to gradually acquire clinical and operative skills, but also to become skillful performers of modern healthcare in general. As these competences are nowadays subject to heightened control and regulations, residents become more alert to their responsibility for service delivery. Consequently, they may become more preoccupied with increasing efficiency at work, dealing with clinical load and work-pressure, than with academic and lifestyle aspects, making them more vulnerable to burnout and intentions to leave the program. Previous studies have suggested that, in order to deal with these challenges and reduce attrition, surgical programs must recruit residents who have the ability to handle such environments [2,27,28,56]. In particular, an important body of literature has explored the relationships between individual traits and attrition in surgical training. For instance, significant attention has been offered to grit, a personal trait referred to perseverance and passion [36,57,58]. Preliminary studies suggested that grit might identify those residents who are at risk for poor psychological well being, but these studies did not reach statistical significance between grit and attrition [36,57]. Despite these findings, a more recent publication reported that grit was correlated with resident’s psychological well-being and attrition in surgical training (measured by the intentions to leave) (58). By using regression analysis, grit was a positive predictor of psychological well-being (B = 0.77, p<0.01), a negative predictor of resident’s depression (B = -0.28, p<0.01) and negative predictor of attrition (B = -0.99, p< .05). However, grit was not a significant predictor of burnout [58]. Studies with a larger number of participants, including different statistical analyses (e.g. path analysis) are required to investigate the associations between grit, burnout and work engagement and attrition. Conversely, in our study we explored a different perspective to understand the resident’s abilities to fit with the environment, instead of personal traits. We demonstrated that job crafting was a strong predictor of work engagement, which, in turn, was inversely related to the intention to leave surgical training. We assume that those residents who have well-developed skills to fit with the environment, are able to engage at work and stay in the program. The findings have been supported by preliminary research indicating the positive relationships of job crafting with performance, at individual and organizational levels [40,41]. The contribution of this study, in sum, is that the intention to leave is a process mediated by residents’ well being at work, which can be molded by their job-crafting abilities. Hence, according to our findings we hypothesize that job crafting appears as an ability to reduce intention to leave. These insights help us better understand the process involved in residents’ retention, thereby approaching the phenomenon from a positive stance.

One of the strengths of this study is its adequate sample size (n = 202) based on the power, bias and a thumb rule indicating at least 100 or 200 observations to conduct SEM analyses [59]. Moreover, the surgical residents in our study population came from different universities, including public and private institutions, and from a wide range of years of training. The response rate of 71% proved sufficient as well. Additionally, we applied instruments that were valid and reliable and translated into Spanish language in accordance with international recommendations. Lastly, we identified a set of plausible hypotheses, which were tested using a robust statistical analysis. A limitation of this study is that we did not measure the structure of the work environment, such as the degree of autonomy residents enjoyed and their involvement in job-related decision-making [60]. Furthermore, the generalizability of our results may be limited as Colombia’s national policies differ from those of other countries. Residents in Colombia must pay a fee to the universities and they work fewer hours in comparison to other countries (e.g. United States). Additionally, it is not easy to obtain a position for surgical training in the country, with the many applicants by far exceeding the number of available positions. The approximate ratio of applicants/position is almost: 20:1 in most programs. We therefore expect that residents are less likely to leave the program, even when working conditions may demand it, because they do not want to waste this unique opportunity in their professional careers, nor the financial resources invested. Hence, the decision to leave the program in this case was potentially influenced by factors external to the working environment. These findings can be generalizable to contexts where the number of surgical positions and funding from government are limited. We therefore invite replication of this research in different contexts.

This study has implications for practice and future research. As for practice, the knowledge that job crafting can improve well-being in highly complex environments where residents combine learning and work may make us want to revisit our coaching strategies. Being aware of this specific job-crafting ability by which job demands (e.g., expectations, making difficult decisions) and job resources (e.g., autonomy, supervision and feedback) can be adapted to the individual’s needs creates opportunities for trainers and trainees alike; for instance, we can incorporate this new insight into interventions aimed at continuous improvement and career development and use it as input for job redesign. In such venture, bearing in mind that most job-crafting skills do not change across the program, the skills to reduce hindering demands (e.g. unrealistic expectations, and mental and emotional intensity at work), deserve particular attention, as they seem to be less well developed. These interventions can add information to the current literature on job crating. In particular more information is required on the effectiveness of job-crafting interventions designed to promote self-directed employee behaviour and to strengthen personal resources [41].

First and foremost, however, we must explore the workplace structure, focusing in particular on the positive and negative value of different demands and resources at the surgical environment. In particular, from our results is essential to explore the value of hindering demands and resources of training (positive and negative) and how they are related to job crafting and, ultimately, to attrition. This exploration could lead to design-specific interventions aimed to boost performance outcomes by improving the relationships between residents and the hindering constituents of the surgical work environment. The effectiveness of such interventions and residents’ receptivity to them requires future research, as do the relationships between the workplace infrastructure and job-crafting behaviors. Similarly, varying degrees of autonomy and supervision, for instance, regulate the extent to which residents are in control of their work, potentially affecting their job-crafting opportunities to reduce hindrances. The role of empowering leadership and job crafting can offer an interesting perspective to explore these relationships in surgical training. Similarly the role of job crafting assessment for selection purposes deserves special attention. Further studies are required on this topic. To conclude, the present study has explored and confirmed the relationship between job crafting, well-being at work, and attrition in surgery. Its findings expose a new view on job crafting as a fundamental ability for dealing with the complex learning and work environment in surgery, creating opportunities for future research.

## Supporting information

S1 File Dataset of the subjects. (XLSX)

## References

[1] Bell RH Jr, Banker MB, Rhodes RS, Biester TW, Lewis FR. Graduate medical education in surgery in the United States. Surg Clin North Am. 2007;87(4):811–23 10.1016/j.suc.2007.06.005 17888781

[2] Ginther DN, Dattani S, Miller S, Hayes P.Thoughts of Quitting General Surgery Residency: Factors in Canada. J Surg Educ. 2016;73(3):513–7. 10.1016/j.jsurg.2015.11.008 26708490

[3] Gifford E, Galante J, Kaji AH, Nguyen V, Nelson MT, Sidwell RA, et al Factors Associated With General Surgery Residents' Desire to Leave Residency Programs: A Multi-institutional Study. JAMA Surg. 2014;149(9):948–53 10.1001/jamasurg.2014.935 25075473

[4] Yeo H, Bucholz E, Ann Sosa J, Curry L, Lewis FR Jr, Jones AT, et al A national study of attrition in general surgery training: which residents leave and where do they go? Ann Surg. 2010;252(3):529–3 10.1097/SLA.0b013e3181f2789c 20739854

[5] Sullivan MC, Yeo H, Roman SA, Ciarleglio MM, Cong X, Bell RH J, et al Surgical residency and attrition: defining the individual and programmatic factors predictive of trainee losses. J Am Coll Surg. 2013;216(3):461–71. 10.1016/j.jamcollsurg.2012.11.005 23266420

[6] Longo WE, Seashore J, Duffy A, Udelsman R. Attrition of categoric general surgery residents: results of a 20-year audit. Am J Surg. 2009:197(6):774–8 10.1016/j.amjsurg.2008.06.038 19178898

[7] Leibrandt TJ, Fassler SA, Morris JB. Attrition and replacement of general surgery residents. Surg Clin North Am. 2004;84(6):1525–35 10.1016/j.suc.2004.06.011 15501273

[8] Maher Z, Milner R, Cripe J, Gaughan J, Fish J, Goldberg AJ. Stress training for the surgical resident. Am J Surg. 2013;205(2):169–74. 10.1016/j.amjsurg.2012.10.007 23331982

[9] Hochberg MS, Berman RS, Kalet AL, Zabar SR, Gillespie C, Pachter H. The stress of residency: recognizing the signs of depression and suicide in you and your fellow residents. Am J Surg. 2013;205(2):141–6. 10.1016/j.amjsurg.2012.08.003 23246287

[10] Rieger A, Fenger S, Neubert S, Weippert M, Kreuzfeld S, Stoll R. Psychophysical workload in the operating room: primary surgeon versus assistant. Surg Endosc. 2015;29(7):1990–8. 10.1007/s00464-014-3899-6 25303917

[11] Jennings ML, Slavin SJ. Resident Wellness Matters: Optimizing Resident Education and Wellness through the Learning Environment. Acad Med. 2015;90(9):1246–50. 10.1097/ACM.0000000000000842 26177527

[12] Bakker AB, Demerouti E, Sanz-Vergel AI. Burnout and Work Engagement: The JD–R Approach Annu. Rev. Organ. Psychol. Organ. Behav. 2014;(1):389–411

[13] Dyrbye LN, Thomas MR, Power DV, Durning S, Moutier C, Massie FS Jr, et al Burnout and serious thoughts of dropping out of medical school: a multi-institutional study. Acad Med. 2010;85(1):94–102. 10.1097/ACM.0b013e3181c46aad 20042833

[14] Dyrbye L, Shanafelt T. A narrative review on burnout experienced by medical students and residents. Med Educ. 2016;50(1):132–49. 10.1111/medu.12927 26695473

[15] Pulcrano M, Evans SR, Sosin M. Quality of Life and Burnout Rates across Surgical Specialties: A Systematic Review. JAMA Surg. 2016 7 13. [Epub ahead of print] 10.1001/jamasurg.2016.1647 27410167

[16] Elmore LC, Jeffe DB, Jin L, Awad MM, Turnbull IR. National Survey of Burnout among US General Surgery Residents. J Am Coll Surg. 2016 5 26. pii: S1072-7515(16)30185-5. 10.1016/j.jamcollsurg.2016.05.014 PMC5476455 27238875

[17] Mache S, Vitzthum K, Klapp BF, Danzer G. Surgeons' work engagement: influencing factors and relations to job and life satisfaction. Surgeon. 2014;12(4):181–90. 10.1016/j.surge.2013.11.015 24325934

[18] Lases SS, Arah OA, Pierik EG, Heineman E, Lombarts MJ. Residents' engagement and empathy associated with their perception of faculty's teaching performance. World J Surg. 2014;38(11):2753–60. 10.1007/s00268-014-2687-8 25008244

[19] Moalem J, Schwartz SI. Three-phase model for surgical training: a proposal for improved resident training, assessment, and satisfaction. J Surg Educ. 2012 Jan-Feb;69(1):70–6. 10.1016/j.jsurg.2011.07.003 22208836

[20] Hom PW, Mitchell TR, Lee TW, Griffeth RW. Reviewing Employee Turnover: Focusing on Proximal Withdrawal States and an Expanded Criterion Psychological Bulletin 2012;138(5) 10.1037/a0027983 22925138

[21] Boswell W, Ren LR, Hinrichs AT. Voluntary Employee Turnover: Determinants, Processess and Future Directions In: Barling J, Cooper C. (Ed) The SAGE Handbook of Organizational Behavior Volume One: Micro Approaches, SAGE Publications, London, 2008.

[22] Ajzen I. The theory of planned behavior. Organizational Behavior and Human Decision Processes, 1991;50(2):179–2

[23] van Dam K. Time frames for leaving. Career Development International, 2008;13(6):560–571.

[24] Everett CB, Helmer SD, Osland JS, Smith RS. General surgery resident attrition and the 80-hour workweek. Am J Surg. 2007;194(6):751–6 10.1016/j.amjsurg.2007.08.033 18005766

[25] Kohanzadeh S, Hayase Y, Lefor MK, Nagata Y, Lefor AT. Factors affecting attrition in graduate surgical education. Am Surg. 2007;73(10):963–6 17983057

[26] Leibrandt TJ, Pezzi CM, Fassler SA, Reilly EF, Morris JB. Has the 80-hour work week had an impact on voluntary attrition in general surgery residency programs? J Am Coll Surg 2006;202(2):340–4. 10.1016/j.jamcollsurg.2005.09.018 16427562

[27] Coverdill JE, Carbonell AM, Fryer J, Fuhrman GM, Harold KL, Hiatt JR, et al A new professionalism? Surgical residents, duty hours restrictions, and shift transitions. Acad Med 2010;85(10 Suppl):S72–S5. 20881709 10.1097/ACM.0b013e3181ed455b

[28] Kelz RR, Mullen JL, Kaiser LR, Pray LA, Shea GP, Drebin JA, et al Prevention of surgical resident attrition by a novel selection strategy. Ann Surg. 2010;252(3):537–1 10.1097/SLA.0b013e3181f27a50 20739855

[29] Tims M, Bakker AB. Job crafting: Towards a new model of individual job redesign. South African Journal of Industrial Psychology, 2010;(36):1–9.

[30] Parker SK, Bindl UK. Proactivity at Work: A Big Picture Perspective on a Construct that Matters In Parker SK, Bindl UK (Eds). Proactivity at Work: Making Things Happen in Organizations. Routledge, UK 2017

[31] Wrzesniewski A, Dutton JE. Crafting a job: Revisioning employees as active crafters of their work. Academy of Management Review, 2001;(26):179–201.

[32] Bakker AB. Top-Down and Bottom-Up Interventions to Increase work engagement APA Handbook of Career Intervention: Vol. 2. Applications, Hartung P. J., Savickas M. L., and Walsh W. B. (Editors-in-Chief) American Psychological Association, USA, 2015.

[33] Albrecht SL, Bakker AB, Gruman JA, Macey W, Saks A. Employee engagement, human resource management practices and competitive advantage. Journal of Organizational Effectiveness: People and Performance, 2015;2(1):7–35

[34] Tims M, Bakker A, Derks D. Development and validation of the job crafting scale. Journal of Vocational Behavior 2012;(80):173–186

[35] Tims M, Bakker A, Derks D, van Rhenen W. Job Crafting at the Team and Individual Level: Implications for Work Engagement and Performance. Group & Organization Management 2013;38(4) 427–454

[36] Burkhart RA, Tholey RM, Guinto D, Yeo CJ, Chojnacki KS. Grit: a marker of residents at risk for attrition? Surgery. 2014;155(6):1014–22. 10.1016/j.surg.2014.01.015 24856121

[37] Howe A, Smajdor A, Stöckl A. Towards an understanding of resilience and its relevance to medical training. Med Educ. 2012;46(4):349–56. 10.1111/j.1365-2923.2011.04188.x 22429170

[38] Doherty EM, Nugent E. Personality factors and medical training: a review of the literature. Med Educ. 2011;45(2):132–40. 10.1111/j.1365-2923.2010.03760.x 21208259

[39] Walker A, Hines J, Brecknell J. Survival of the Grittiest? Consultant Surgeons Are Significantly Grittier Than Their Junior Trainees. J Surg Educ, Vol. 73, Issue 4, p730–734 10.1016/j.jsurg.2016.01.012 27025568

[40] Lombarts KM, Heineman MJ, Scherpbier AJ, Arah OA. Effect of the Learning Climate of Residency Programs on Faculty's Teaching Performance as Evaluated by Residents. PloS one. 2014;9(1):e86512 10.1371/journal.pone.0086512 24489734 PMC3904911

[41] Plomp J., Tims M., Akkermans J., Khapova S.N., Jansen P.G.W, & Bakker A.B. Career competencies and job crafting: How proactive employees influence their well-being. Career Development International, 2016: 21, 587–602.

[42] Le Blanc P.M., Demerouti E., & Bakker A.B. How can I shape my job to suit me better? Job crafting for sustainable employees and organizations In Chmiel N., Fraccaroli F. & Sverke M. (Eds), An introduction to work and organizational psychology: An international perspective (3rd edition; pp. 48–63). New York: Wiley, 2017.

[43] Wasserman MA. A Strategy to Reduce General Surgery Resident Attrition: A Resident's Perspective. JAMA Surg. 2016 3;151(3):215–6. 10.1001/jamasurg.2015.4607 26650772

[44] Ficapal-Cusí P, Torrent-Sellens J, Boada-Grau J, Hontangas-Beltrán P. “Job change without changing job? Exploring job crafting in Spain” [online working paper]. (Working Paper Series; WP14- 005). IN3 Working Paper Series. IN3 (UOC) 2014. [Accessed: 10/05/2017].<http://journals.uoc.edu/ojs/index.php/in3-working-paper-series/article/view/n14-ficapalcusi-torrent-sellens-boada-grau-hontangasbeltran/n14-ficapal-cusi-torrent-sellensboada-grau-hontangas-beltran>

[45] Yang Y, Liu YH, Liu JY, Zhang HF. The impact of work support and organizational career growth on nurse turnover intention in China. International Journal of Nursing Sciences, 2015;2(2): 134–139

[46] Bullinger M, Alonso J, Apolone G, Leplège A, Sullivan M, Wood-Dauphinee S, et al Translating health status questionnaires and evaluating their quality: the IQOLA Project approach. International Quality of Life Assessment. J Clin Epidemiol. 1998;51(11):913–23. 9817108 10.1016/s0895-4356(98)00082-1

[47] Shaufeli WB, Bakker A. Work engagement Scale. Preliminary Manual. Occupational Health Psychology Unit Utrecht University, 2003 Available in: http://www.beanmanaged.com/doc/pdf/arnoldbakker/articles/articles_arnold_bakker_87.pdf

[48] Maslach C, Schaufeli WB, Leiter MP. Job burnout. Annu Rev Psychol 2001;52:397–422. 10.1146/annurev.psych.52.1.397 11148311

[49] Maslach C, Jackson SE, Leiter MP. Maslach Burnout Inventory. Manual (3rd ed.). Palo Alto, CA: Consulting Psychologists Press, 1996.

[50] Kirk RE. Practical significance: A concept whose time has come. Educational and psychological measurement, 1996;(56):746–759

[51] Hatcher L. Advanced statistics in research Shadow Finch Media, USA, 2013

[52] Cohen J. Statistical power analysis for the behavioural sciences (2nd Ed). Hillsdale, NJ: Lawrence Erlbaum Associates, 1998.

[53] Hauer KE, Ten Cate O, Boscardin C, Irby DM, Iobst W, O'Sullivan PS. Understanding trust as an essential element of trainee supervision and learning in the workplace. Adv Health Sci Educ Theory Pract. 2014 8;19(3):435–56. 10.1007/s10459-013-9474-4 23892689

[54] Ten Cate O Hart D, Ankel F, Busari J, Englander R, Glasgow N, et al Entrustment Decision Making in Clinical Training. Acad Med. 2016 2;91(2):191–8. 10.1097/ACM.0000000000001044 26630606

[55] Crommelinck M, Anseel F. Understanding and encouraging feedback-seeking behaviour: a literature review. Med Educ. 2013 3;47(3):232–41 10.1111/medu.12075 23398009

[56] Louridas M, Szasz P, Montbrun S, Harris KA, Grantcharov TP. Optimizing the Selection of General Surgery Residents: A National Consensus. J Surg Educ. 2016 7 28. pii: S1931-7204(16)30091-5. 10.1016/j.jsurg.2016.06.015 27476793

[57] Salles A, Cohen GL, Mueller CM. The relationship between grit and resident well-being. Am J Surg. 2014 2;207(2):251–4 10.1016/j.amjsurg.2013.09.006 24238604

[58] Salles A, Lin D, Liebert C, Esquivel M, Lau JN, Greco RS, Mueller C. Grit as a predictor of risk of attrition in surgical residency. Am J Surg. 2017 2;213(2):288–291. 10.1016/j.amjsurg.2016.10.012 27932088

[59] Wolf EJ, Harrington KM, Clark SL, Miller MW. Sample Size Requirements for Structural Equation Models: An Evaluation of Power, Bias, and Solution Propriety. Educational and psychological measurement. 2013;76(6):913–934. 10.1177/0013164413495237 25705052 PMC4334479

[60] O'Driscoll MP, Pierce JL, Coghlan AM. The Psychology of Ownership: Work environment structure, organizational commitment and citizenship behaviours. Group & Organization Management, 2006;31(3): 388–41

