# Correction: Taking control: Is job crafting related to the intention to leave surgical training?

**DOI:** 10.1371/journal.pone.0202293

**Published:** 2018-08-08

**Authors:** Luis Carlos Dominguez, Laurents Stassen, Willem de Grave, Alvaro Sanabria, Edgar Alfonso, Diana Dolmans

## Notice of republication

An incorrect version of S1 File was published in error. This article was republished on July 31, 2018 to correct for this error. Please download this article again to view the correct version.

## References

[1] Dominguez LC, Stassen L, de Grave W, Sanabria A, Alfonso E, Dolmans D (2018) Taking control: Is job crafting related to the intention to leave surgical training? PLoS ONE 13(6): e0197276 10.1371/journal.pone.0197276 29856750 PMC5983422

